# Beliefs surrounding the use of inhaled asthma medication in The Gambia: a qualitative study of asthma patients and healthcare workers

**DOI:** 10.1038/s41533-024-00390-x

**Published:** 2024-10-17

**Authors:** S. Jayasooriya, M. Inoue, H. Allen, M. Bojang, A. Ceesay, S. Touray, R. Cooper, K. Mortimer, J. Balen

**Affiliations:** 1 School of Medicine and Population Health, University of Sheffield, Sheffield, UK; 2 Department of Geography, University of Sheffield, Sheffield, UK; 3 Medical Research Council, The Gambia at London School of Tropical Medicine and Hygiene, Fajara, The Gambia; 4 Permian Health Lung Institute, The Woodlands, Texas USA; 5 Cambridge Africa, Department of Pathology, University of Cambridge, Cambridge, UK; 6 Department of Paediatrics and Child Health, School of Clinical Medicine, College of Health Sciences, University of KwaZulu-Natal, Durban, South Africa; 7 Respiratory Medicine, Liverpool University Hospitals, NHS Foundation Trust, Liverpool, UK; 8 School of Allied and Public Health Professions, Canterbury Christ Church University, Canterbury, UK

**Keywords:** Asthma, Health care

## Abstract

Asthma-related mortality is high in low- and middle-income countries. Little is known about public perceptions of inhaled medicines. We conducted semi-structured interviews with asthma patients and healthcare workers at three secondary care facilities in The Gambia, between August and November 2022. Thematic analysis was used to interpret these data. A total of 20 patients and 15 healthcare workers were interviewed. Both groups noted limited access to inhalers was an issue resulting in continued use of oral medications. Some patients recognised the benefits of inhalers, yet beliefs that inhalers were dangerous were common. Reliance on oral short-acting beta agonists meant patients saw asthma as a recurrent acute condition resulting in an emphasis on hospital management with little awareness of inhaled preventative medicines. Increasing access to inhaled medicines has the potential to reduce costly avoidable admissions, but socio-cultural factors, in addition to medication supply, need addressing.

## Introduction

Asthma is one of the most common non-communicable diseases among adults and is estimated by the Global Burden of Disease (2019) to affect an estimated 262 million people globally^1–4^. The highest asthma-related mortality is found in low- and middle-income countries (LMICs)^5^. Effective inhaled medicines for chronic respiratory diseases such as asthma are known to be both difficult to obtain and poorly utilised in sub-Saharan Africa^6–8^. Data from The Gambia, West Africa, has highlighted the lack of affordable access to inhalers, with combination inhalers (corticosteroid and fast/long-acting beta2 agonists, e.g., budesonide and formoterol) being the least accessible^9^. Combination inhalers are on the World Health Organization’s (WHO) essential medicines list for the treatment of asthma, and the Global Initiative for Asthma (GINA) recommends as-needed combination low-dose inhaled corticosteroid-formoterol as a first step in asthma management while recognising the importance of access to inhalers, adherence and inhaler technique^10^.

In most LMICs, there is a lack of national guidance for asthma care, and diagnostic testing for asthma is not universally available^11^. The limited access to WHO’s essential medicines for asthma in countries such as The Gambia makes the implementation of international asthma guidance and diagnostic testing a significant challenge^9,12^. Coupled with the lack of accessibility to inhaled medicines for the effective treatment of asthma, in some LMICs, including The Gambia, there has been continued use of oral short-acting beta agonist (SABA) tablets^12,13^. The continued use of oral SABA adds a further dimension of complexity when trying to implement appropriate guidance for asthma treatment. Clinical standards have been developed using a Delphi process by those working in LMIC settings to offer a pragmatic approach to asthma treatment, specifically recognising settings where WHO essential medicines are unavailable^14^. Alongside providing clinical standards for a pragmatic approach to asthma care, utilising available medications, it strongly advocates for wider system change to improve country access to inhaled medicines.

Reasons for the limited availability and use of inhalers are complex, not fully understood and relate to multiple levels and barriers within a health system^15^. This includes the influence of service users’ and healthcare workers’ perceptions and experiences of inhaled versus oral medicine, which has been explored in relation to oral versus inhaled corticosteroids in a high-income setting but, to the best of our knowledge, has not been explored in an LMIC setting^16^. This study explores patients’ and providers’ perceptions and lived experiences of inhaled medicines for asthma care, in line with current GINA recommendations, in The Gambia, where there is limited access to inhaled medicines.

## Methods

### Study design and setting

This qualitative phenomenological study investigated the lived experience of patients with asthma, and healthcare workers in The Gambia. It focuses on how individuals perceive and make sense of their experiences, from their own perspectives. The study was conducted in three secondary care health facilities in The Gambia, namely, Kanifing General Hospital, Edward Francis Small Teaching Hospital Bakau Centre (formally Ndemban Clinic) and the Medical Research Council Unit The Gambia at LSHTM Clinic (MRC Clinic). The former two are government-funded, while the latter, the MRC clinic, is predominantly a research clinic, often employing visiting research staff from high-income settings. While patients from each of the three facilities were included, healthcare workers from the MRC clinic were not included as they were, not government employees.

### Participant recruitment

The study used purposive and snowball sampling to recruit participants. Frontline healthcare workers, including doctors, nurses and pharmacists working in the two government-funded secondary care health facilities, were identified and invited to participate in the study. Interviews were then scheduled and conducted in their places of work at convenient times. Interviews lasted between 60 and 90 min.

Patients with symptoms consistent with an acute exacerbation of asthma in the emergency departments and in-patient wards were approached once clinically stable, with the assistance of clinical staff. If symptoms were mild patients were approached once identified. Patients experiencing severe exacerbations were not excluded and were approached and interviewed downstream once clinically stable. Interviews were conducted in hospitals and home settings. Symptoms considered consistent with asthma were used in recognition of the limited access to respiratory diagnostic testing in The Gambia, and hence, many patients in this setting lack a formal diagnosis. The defining symptoms used were, as per GINA guidance, shortness of breath, wheezing, chest tightness and cough, and patients were only approached if deemed appropriate by the clinical staff overseeing the sampling.

### Ethics

Ethical approval was obtained from the Medical Research Council The Gambia at the London School of Tropical Medicine and Hygiene Ethics Committee Ref: 26173 and approved by the University of Sheffield Ethics Committee Ref: 709. All ethical standards and procedures were met, including processes for informed consent of study participants.

### Data collection and analysis

Semi-structured interviews were conducted between the 1st of August and the 30th of November 2022, by trained female Gambian social scientists (H.A., M.B., and A.C.) in English, Wolof or Mandinka, as chosen by the participants.

Prior to this research project H.A., M.B. and A.C had no personal experience of asthma within the Gambian health system. This enabled an open approach to interviewing with the participant as the central expert but also had the potential to limit the depth of exploration. To mitigate this basic training on asthma care, study processes and procedures was provided by S.J., a female general practitioner with a special interest in respiratory medicine, with weekly scheduled reflective meetings throughout the data collection period. As part of this process H.A. joined a stakeholder meeting, including World Health Organization representation, on access to inhaled medicines for asthma prior to study initiation^7^.

Semi-structured topic guides (Appendix 1) were used to facilitate the interviews, and these were conducted until there were no new themes emerging, and therefore data saturation was reached. Interviews were recorded, and for the purpose of reporting, they were anonymised and transcribed in English by experienced translators (M.B. and A.C.). All transcriptions were repeatedly read and re-read by S.J. and M.I. to enable familiarisation with the data and independently coded using NVivo v14 data management software. Inductive analysis was used to generate codes. After the initial coding of interview data, initial themes were generated, which were then refined using an iterative approach and finalised through discussions within the study team at regular intervals^17,18^. These permitted input and interpretation by all team members, with all inputs considered equally, to avoid bias. Comparisons made were reflected on during generation of themes between patients, healthcare workers, and settings to provide an element of triangulation and credibility and determine the trustworthiness of these data^19^. A workshop was held in April 2024 where findings were presented and discussed with participants with no further modifications arising.”

## Results

All eligible healthcare workers agreed to participate in the study, and only one patient participant who was approached declined. A total of 20 patients (12 females) and 15 healthcare providers (five nurses, five pharmacists and five doctors, 10 of which were male) were interviewed (Table 1).

**Table 1:** Characteristics of healthcare workers and patients.

	Healthcare workers (*n* = 15)	Patients (*n* = 20)
*Age (years)	31 (26–41)	43 (27–69)
Sex ratio (M:F)	2:1	2:3
Time post-qualification (years)	7 (3–17)	-
Professions	3 Pharmacists (1 head Pharmacist) Pharmacy technician Director of National Pharmaceutical Services 5 Nurses (3 senior nurses) 5 Doctors (1 head of Accident and Emergency)	-

*Age and time post-qualification are shown as median (range).

All but three patients were recruited from Kanifing General Hospital and Edward Francis Small Teaching Hospital Bakau Centre (formally Ndemban Clinic), and the final three patients were recruited from the Medical Research Council Gambia Clinic at LSHTM in The Gambia. Health professionals were recruited from the two government sector healthcare facilities, six from Kanifing General Hospital and nine from Edward Francis Small Teaching Hospital Bakau Centre (formally Ndemban Clinic).

Patients and providers, while positively recognising the benefits of inhaler use for asthma, expressed concern about the lack of access to inhaled medicines in The Gambian health system and noted this was a major challenge to asthma care. Both patients and providers had positive perceptions of inhaler use for asthma despite affordability and availability issues, and patient perceived harms from inhaler use. Patient experiences of inhaler use overwhelmingly focused on the use of inhaled SABA, as they had little or no experience with the use of inhaled corticosteroids for the treatment of asthma. The limited supply and lack of inhaled corticosteriod use meant few patients experienced the benefits of preventative treatment. Continued use of cheap oral SABA reinforced perceptions of asthma as a recurrent acute illness rather than a chronic condition that is often life-long. The requirement for ongoing asthma treatment and the ability to avoid acute attacks that lead to admissions was notably absent. Table 2 lists the themes and subthemes identified.

**Table 2:** Themes.

Theme	Subthemes	
	Healthcare workers	Patients
Poor availability and affordability affect prescribing and use of inhaled medicines	Unable to prescribe, as inhaled medicines often unavailable or out of stock	Lack of (diagnostic) testing and limited understanding of asthma
	Preferentially prescribing tablets	Regular use of oral salbutamol
	Difficulty advising on use of inhalers due to lack of availability	Lack of availability of inhalers to use
	High cost of asthma medication	Use of private pharmacies at high cost
Perceived harms of inhaled medicines	Patient fears of use of inhalers and addictive nature	Fears of long-term use of inhaled medications
	Concern of harm once used and then no longer unavailable	Illness is seen as God’s will and recovery in God’s hands
Asthma as an acute and not chronic condition leading to recurrent hospitalisation	Multiple hospital visits that could be avoided	Inevitability of visits to hospital on a regular basis
	Same regular patients recurrently seen in hospital settings	Inhaled medicines enable travel to a hospital
	Acceptance of nebulisers and injectable treatment but recognition that this is suboptimal	Aware that symptoms will reoccur and a limited sense that this could be avoided or managed

### Poor availability and affordability affect prescribing and use of inhaled medicines

Patients repeatedly echoed experiences of being unable to obtain inhaled medications both in the public sector and in private sector pharmacies:

“[…] *let’s be frank to each other, public hospitals don’t have medications. When you go there you wouldn’t have medications because none of them have medications […]”* SU08

“[…]*You know not everyone have the money to buy inhalers so if they give them the salbutamol pills they will take it and will not buy the inhaler*. *[…]”* SU17

What was very evident was that in contrast to the difficulties accessing inhalers, there was relatively easy access to SABA tablets, and these were often obtained from the public sector hospital:

*“[…] at the hospital they will prescribe salbutamol for me take one tablet three times a day […]” SU05*

These findings from patient interviews were mirrored in health professional interviews, with many reflecting on the lack of availability of inhaled medicines, even if prescribed:

*“[…] all that I can say is they* [inhaled medicines] *are not available; for most they are not available.”* HP03

*“[…] as I told you in the government sector, and you don’t have much in the supply chain with inhale medicines.”* HP10

There was a sense of futility and passive acceptance among healthcare workers, and they noted that their general prescribing behaviours were influenced by the availability of medicines. In many instances, practitioners were driven towards the prescribing of SABA tablets due to a lack of access to inhalers in the public sector:

*“We, like I said, we have the oral, oral medicines in our health system. So basically, I desperately use the oral medications instead of inhaled medicines.”* HP05

*“For here, what we normally prescribe more, let’s say maybe 80% or more, is salbutamol tablets that that I’ve seen more.”* HP14

### Perceived harms of inhaled medicines

Patients and practitioners both described similar strongly held beliefs that they had encountered and which were said to be widely held by community members regarding the use of inhaled medicines.

*“But when my husband asked me what did they give you, so I showed him, and he asked me not use the inhaler because I don’t have breathlessness frequently and if you get used to it, it might be a problem.”* SU20

The beliefs encountered reflected concerns about dependence and worsening of asthma symptoms due to treatment, combined with, in some instances, the fear of death if inhaled medications were used and subsequently became unavailable. These beliefs were said to be often expressed by those within patients’ social networks. These harms were felt to be widespread:

*“….like if I am addicted to and if I don’t have it, I can die… but some of the comments I receive from people, people are afraid of addictions, getting addicted to it and not getting it at the time of need so that is a concern people have. They are afraid of getting addicted to it.”* SU16

Health professionals also reported this belief among their patients, and they noted that similar ideas and notions appeared to be strongly held and pervasive within Gambian society

*“So sometimes they have this notion that maybe I am going to die because I must use the inhaler. Someone told me that if you stop using inhaler you will die.”* HP01

*“….most patient will not use because they think it makes you glue to it and because you glue to it you don’t recovery and you tend to have it a habit, that’s one misconception of it.”* HP06

However, some patients directly challenged the circulating perceived harms based on their personal experiences of use of inhaled medicines.

*“They said if one use it the person don’t recover from the illness but that doesn’t discourage me because each and every person know how one feels when sick. If the inhaler is helpful, the one using it knows about it. So those mere words don’t discourage me and there is someone with asthma who gave me his inhalers because he don’t use it.”* SU12

*“Many people do tell me not to get so used to the inhaler but I am still using it because it makes me feel relief.”* SU13

### Positive perceptions of asthma inhalers over tablets

Despite the lack of access to inhaled medicines and persistent negative lay beliefs said to be circulating in the community, patients and practitioners had positive perceptions of inhaled medicines for the treatment of asthma. This was apparent from the descriptions patients made by directly comparing tablets and inhalers:

*“…..the inhaler* [is] *quicker to feel relief than the pills.”* SU02

*“The inhaler* [is] *the* [more] *quick reliever than the tablets because if I take the tablets I do feel relief but it takes time.”* SU05

*“….when you have attack and have the inhaler with you, you just use it and feel relief…”* SU18

All healthcare workers interviewed expressed positive attitudes towards inhaler use and recognised their value.

*“It (inhaler) works much better than oral. Because they are giving at a point of action where they need to act”* HP06

Healthcare workers did describe a reluctance around the prescribing of inhalers, as they are harder to source and pay for in addition to circulating beliefs around their addictive potential and risk of death.

*“but if they are not available patients have to buy it from outside which is an obstacle because some of them cannot afford it”* HP07

### Asthma as an acute and not chronic condition leading to recurrent hospitalisation

There was a relative absence of the role of prevention in asthma, and the dominant discourse was that this was an acute condition and, because of this, a view that hospital management was the most appropriate way to treat asthma. Only two healthcare workers reflected on their experiences of the use of inhaled corticosteroid, with one noting how a lack of availability reinforces patients’ limited awareness of the need for it:

*“Once they* [patients] *start these, these daily, like inhaled corticosteroid, the difference* [is] *remarkable……*……*so basically, the mainstay of treatment is inhaled corticosteroids. For many years, it’s not been available, so* [patients], *they’re not aware of it.”* HP04

Perhaps not surprisingly given the lack of access, the use of inhaled corticosteroids was a notable omission from patients’ narratives and those of most healthcare workers. Asthma was overwhelmingly described by patients as an acute illness of repeated attacks, and there was no reference in patient narratives to it being a long-term chronic condition. Moreover, the acute perception of asthma led to the dominance of the hospital being the most appropriate health setting within which to manage asthma:

*“I have been going to different hospital whenever I have attack. I went to many hospital because I have asthma since I was a child.”* SU01

*“….and when I have attack I will go to Kanifing General Hospital and they will nebulize me and go home. It will take a month or three weeks it comes back again and go back to the hospital again.”* SU05

*“Sometimes will get back home and within 30 to 40* *minutes will go back again to the hospital so my condition was not improving*…” SU17

This was also reflected in the descriptions by healthcare workers of their experiences of treating asthma patients in the health system:

*“Like I said, like if you if you spend a week at our emergency department, you see the same faces come back forth.”* HP04

*“but when it comes to dealing with these patients, we manage them all the time*—*they keep going and coming* [back]*.”* HP09

The limited supply and lack of inhaled corticosteriod use means few patients experience the benefits of preventative treatment. This, coupled with the continued use of cheap oral SABA, reinforces the perception of asthma as a recurrent acute illness.

## Discussion

This study demonstrates that patients living with asthma in The Gambia have generally positive attitudes towards inhaler use. This is despite overarching reference to their continued lack of availability, ongoing use of short-acting beta agonist tablets, and broadly perceived harms of inhaled medicines circulating within Gambian society. Patients’ views were supported by the experiences of most healthcare workers, in terms of the limited availability and use of inhaled medicine, pervasive lay beliefs about their perceived harms, and patterns of health service use (i.e., recurrent hospitalisation for acute exacerbations of asthma).

Both patients and healthcare workers focused on the acute nature of asthma and described repeated hospital attendances. This was due to limited supply and lack of use of inhaled medicines alongside the continued use of cheap oral SABA. There was silence in the data on the part of patients in terms of awareness of inhaled corticosteroid use, the idea that hospital attendance could be avoidable and that asthma is a long-term chronic condition. The cost of treating asthma episodically in a hospital setting rather than with inhaled corticosteroids delivered in the community is extremely high, both economically and, also in terms of the human cost, with greater mortality rates likely to be seen^20^.

It is known that access to inhaled medicines is limited in LMIC settings with prohibitive cost implications in The Gambia^6–9^. This study’s findings are in line with those of patients where medicine stockouts are a problem and similar coping strategies have been noted, such as purchasing from local chemists and admitting oneself to an in-patient facility^21^. Previous international initiatives to improve access to inhaled medicines, such as the Union Asthma Drug Facility, have historically been unable to address this issue. One of the persistent challenges is that countries do not recognise the implications of not prioritising access to inhaled medicines for chronic respiratory diseases such as asthma^22^. Without Ministry-level recognition of this issue, efforts to improve access to inhaled medicines through simplified procurement mechanisms are likely to fail.

This work demonstrates that despite significant contextual barriers, in a setting with very limited access to inhaled medicines, their value is still recognised by those living with asthma and the healthcare workers treating them. This suggests that policy changes to prioritise and improve access to inhaled medicines for asthma would be well received by patients and providers but that there is also a need to normalise inhaler use in parallel with policy change. Socio-cultural factors have previously been shown to influence adherence to treatments, for example, for malaria in The Gambia^23^.

There are some notable limitations to this study. First, the patients and healthcare workers were predominantly from a secondary care setting and therefore represent a specific voice and highlight the embodied experiences of those already accessing conventional care in the public health system. Participant selection was limited by the fact that patients who access only community services or traditional medicine without approaching a secondary care setting were not captured here. These patients may hold very different attitudes towards the use of inhaled medication. However, arguably, patients accessing acute hospital-based care are likely to be those that are at the severe end of the asthma spectrum and therefore face both a higher risk of complications, including death, and can be the most costly to treat. Therefore, understanding their perspectives on inhaler use is essential. Second, interviews were conducted in local languages and then transcribed into English. There will inevitably be potential for misinterpretation, to mitigate for this both interviewing and transcribing was conducted by multi-lingual Gambian experienced social scientists, bringing with them an in-depth understanding of the cultural context and local understanding of language used.

Further research including a more generalisable survey or economic modelling would assist in demonstrating cost implications and in-country gains from changes in policy and clinical practice in The Gambia, and given the overwhelming internationally available body of evidence for changing practice^10,11,14,15,20,24–28^, this could be significant for the Gambian setting. This work advocates for health system change both locally in The Gambia, but also as an example by illustrating an important issue relevant to other LMICs.

In clinical settings, the use of inhaled medications for asthma care should be encouraged, specifically as a long-term treatment that can prevent/reduce exacerbation frequency. This work demonstrates that this would be a good use of resources. At policy level access to inhaled medicines should be prioritised, and resources provided to inform both the workforce and patients on the importance of asthma care and high-quality treatment.

## Conclusions

This research highlights the lack of access to and persistence of lay beliefs about the perceived harms of inhaled medicines for asthma. There is a pressing need for change to improve asthma care as well as a receptiveness to this from patients and healthcare workers. Prioritised affordable access to inhaled medicines for the treatment of asthma, could have a significant impact on the lived experiences of people with asthma in terms of reduced morbidity and mortality, as well as reductions in healthcare seeking. This would save lives and reduce avoidable repeated acute admissions, which represent a huge cost to health systems. Ultimately increasing inhaler access has the potential to save lives, but socio-cultural factors, in addition to medication supply barriers, need to be addressed.

## Supplementary information

Supplementary Information

## Data Availability

These interview data that support the findings of this study are held by the University of Sheffield within a password-protected repository and can be shared following a request to the corresponding author.
