# Supplementary material for: Beliefs surrounding the use of inhaled asthma medication in The Gambia: a qualitative study of asthma patients and healthcare workers
Source: NPJ Prim Care Respir Med. 2024 Oct 17;34:29. doi: 10.1038/s41533-024-00390-x (PMC11487242; doi:10.1038/s41533-024-00390-x)
Supplement: Supplementary file 1 — Supplementary Information [file 41533_2024_390_MOESM1_ESM.docx]

Appendix 1: Semi-structured interview guides

**Theme Guide for in-depth Health Professional interviews**

**Introduction**

Ground rules, consent, any questions, appropriate icebreakers to start discussion

**Part A: Experience**

- What is your role and position at this facility and did this change during the Covid pandemic?
  - Where did you go to medical school and when did you qualify?
  - How many years are you post qualifying? Have you completed any postgraduate qualification?
- Can you tell me about your professional experience particularly related to asthma and chronic respiratory diseases?
  - What motivated you to do this?
  - How long have you been doing this?
- Did you have any exposure to the use of inhaled medicine(s), such as Beclomethasone or Ventolin, during your training?
- Probe, in relation to:
  - Formal exposure during training programmes?
  - Personal lived experience?
- Do you have access to any current guidance and/or support for the prescribing of inhaled medication in your current health professional role?

**Part B: Prescribing experience**

- In your current health professional role what is your current experience of use of inhaled medicines(s), such as Beclomethasone or Ventolin?
- Probe, in relation to:
  - Availability – are they always available? Accessible? Cost?
  - Acceptability?
    - How do patients react to being prescribed inhaled medicines?
    - Have any patients ever refused? Why? Do you follow up with them or do you just leave them?
  - What are your reflections on the quality of the inhaled medications available? Are there any problems with the expiry dates?
- How able do you feel teaching people (patients and their family members) how to use these inhaled medicine(s)?
- Probe, in relation to:
  - Practicalities? Space (where would you do that/see patients)? Time? Support?

**Part C: Beliefs around inhaled medicine(s)**

- What is your opinion of inhaled medicine(s) such as Beclomethasone or Ventolin?
- How well do you think this works compared to oral medicines?
- How able do you consider your patients to use these inhaled medicine(s) such as Beclomethasone or Ventolin?
- Do you think there is any benefit to patients using inhaled medicine(s) such as Beclomethasone or Ventolin? Inhaler devices? Spacers? Nebulisers?
- Do you think traditional/herbal medicines could effectively treat or manage asthma?

**Part C: Barriers to usage**

- What do you think other professionals consider as the main problems that prevent use of inhaled medicine(s) for patients with chronic respiratory problems such as asthma?
- What do you think about this?

**Part D: Improving use of inhaled Medicine(s)**

- What do you think would improve use of inhaled medicine(s) in The Gambia?
  - What are some of the changes you’d like to see?
- What are your thoughts on:
  - Improving diagnostic testing for chronic respiratory diseases?
  - Health system investment – staff and medication?
  - Training?
  - Sensitization and education?
  - Drug company advertising/promotion?
- What do you think would be most important out of these and most likely to bring about positive change? Which would influence your practice in any way and how?

**Closing questions and thanks!**

- Are there any things we haven’t asked you which you feel might be important to discuss here?
- Do you have any (further) questions for me/us?
- Thank you very much for your time and inputs today!

**Theme Guide for in-depth patient interviews**

**Introduction**

Ground rules, consent, any questions, appropriate icebreakers to start discussion

**Part A: Experience of health care**

- What treatments have been you been given for your breathing difficulties?
- Probe, in relation to whether any differences at:
  - Rural clinics?
  - District or general hospitals?
  - Private pharmacies?
- What guidance and/or support did you receive from health professionals for use of any treatments you were given for your chronic respiratory symptoms?
- What do you understand about the symptoms you experience such as breathlessness and what is causing them?

**Part B: Prescribing experience**

- What is your experience of use of inhaled medicine(s), which are medicines you breath in you don’t eat, such as Beclomethasone or Ventolin?
- Probe, in relation to:
  - Accessible? Cost?
  - Acceptability?
  - Quality? Expiry dates?
- How able did you feel to use these inhaled medicine(s) if prescribed?
- Probe, in relation to:
  - Duration?
  - Understanding of what to do?

**Part C: Beliefs around inhaled medicine(s)**

- What, is your opinion, of inhaled medicine(s) such as Beclomethasone or Ventolin?
- How well do you think this works compared to oral medicines?
- Do you think there is any benefit to patients using inhaled medicine(s) such as Beclomethasone or Ventolin? Inhaler devices? Spacers? Nebulisers?

**Part C: Barriers to usage**

- What are the main problems that prevent the use of inhaled medicine(s) for patients with chronic respiratory problems such as asthma, according to you?
- How do you think other people in your community think about this?

**Part D: Improving use of inhaled Medicine(s)**

- What do you think would improve the use of inhaled medicine(s) in The Gambia?
- What are your thoughts on:
  - Improving diagnostic testing for chronic respiratory diseases?
  - Health system investment – staff and medication?
  - Training?
  - Public engagement and education?
  - Drug company advertising/ promotion?
- What do you think would be most important out of these and most likely to bring about change? Which would influence you?

**Closing questions and thanks!**

- Are there any things we haven’t asked you which you feel might be important to discuss here?
- Do you have any (further) questions for me / us?
- Thank you very much for your time and inputs today!
